# Particularidades dos Pacientes com Arterite de Takayasu em Idade Mais Avançada: Estudo Coorte, Retrospectivo e Transversal

**DOI:** 10.36660/abc.20220463

**Published:** 2022-12-20

**Authors:** João Calvino Soares de Oliveira, Alexandre Moura dos Santos, Mariana Freitas de Aguiar, Jucier Gonçalves, Alexandre Wagner Silva de Souza, Rosa Maria R. Pereira, Samuel Katsuyuki Shinjo

**Affiliations:** 1 Hospital das Clínicas Faculdade de Medicina Universidade de São Paulo São Paulo SP Brasil Divisão de Reumatologia – Hospital das Clínicas HCFMUSP – Faculdade de Medicina – Universidade de São Paulo, São Paulo, SP – Brasil; 2 Divisão de Reumatologia Universidade Federal de São Paulo São Paulo SP Brasil Divisão de Reumatologia – Universidade Federal de São Paulo – UNIFESP, São Paulo, SP – Brasil; † In memorium

**Keywords:** Envelhecimento, Vasculite Sistêmica, Doenças da Aorta

## Abstract

**Fundamentos:**

Poucos estudos avaliaram pacientes idosos com Arterite de Takayasu (AT).

**Objetivo:**

Avaliar o progresso de AT em diferentes grupos etários em seus possíveis efeitos sobre o tratamento medicamentoso e atividade da doença.

**Métodos:**

este estudo transversal, retrospectivo, do tipo coorte incluiu 66 pacientes com AT. Os pacientes foram entrevistados, e dados dos 12 meses anteriores foram coletados dos prontuários médicos eletrônicos. Os pacientes foram divididos em quatro quartis de acordo com idade atual, e comparados quanto aos dados clínicos e laboratoriais, tratamento, comorbidades, status da doença, e status funcional. Um p<0,05 foi estabelecido como estatisticamente significativo.

**Resultados:**

Os grupos foram definidos como Q1(22-36 anos, n=16), Q2(37-42 anos, n=18), Q3(43-49 anos, n=17), e Q4(51-66 anos, n=15). A frequência de pacientes com atividade da doença, fadiga, comorbidades e comprometimentos vasculares, e o índice de extensão da doença (DEI. Tak) foram comparáveis entre os grupos. Pacientes com idade mais avançada apresentaram maior duração da doença (p=0,001) e maior comprometimento do status funcional (Q2 versus Q3, p=0,003); menos pacientes usaram prednisona (Q1:43,8%; Q2:33,3%; Q3:11,8%; e Q4:6,7%; p=0,049) e agentes imunossupressores [Q1:100,0%; Q2:66,7%; Q3:58,8% e Q4:46,7%; Q1 *versus* Q3 (p=0,043) e Q1 *versus* Q4 (p=0,005) nas análises *post-hoc* ]. Além disso, os níveis de danos da doença, sintomas de uma nova ocorrência de AT, e complicações nos 12 meses precedentes não foram diferentes entre os grupos.

**Conclusão:**

Pacientes com AT e idade mais avançada requerem mínima intervenção medicamentosa e apresentam maior comprometimento no status funcional, o que pode ser atribuído a fatores relacionados ao envelhecimento.

## Introdução

A Arterite de Takayasu (AT) é uma vasculite sistêmica primária que afeta preferencialmente vasos de calibres maiores, tais como a artéria aorta e suas ramificações próximas. O início da doença ocorre mais comumente em mulheres com idade inferior a 40 anos.^1 , 2^

A AT é clinicamente caracterizada por períodos alternados de atividade e remissão, refletindo diretamente os vários estados inflamatórios dos vasos afetados.^3 - 5^ Cerca de 20% dos pacientes apresentam um curso monofásico, autolimitado, com sintomas constitucionais que podem não ter repercussões clínicas. No entanto, os demais pacientes podem apresentar inflamação vascular progressiva ou um curso recorrente grave, com manifestações clínicas que variam de acordo com o território vascular afetado, claudicação de membros, síncope, dor torácica, hipertensão renovascular, e pulsos reduzidos/ausentes.^6 - 8^

Contudo, na prática clínica, caracterizar a atividade da doença é muitas vezes difícil, uma vez que a inflamação das paredes dos vasos nem sempre reflete ou segue outas manifestações inflamatórias sistêmicas ou aumento de proteínas de fase aguda.^9^ Por outro lado, uma história detalhada, avaliação física, e achados de imagens podem ajudar a definir o estado da doença.^10^ Ainda, apesar de suas limitações, há critérios para avaliar a atividade da doença na AT, como o *Indian Takayasu Clinical Activity Score* (ITAS2010) e o *escore* do *National Institute of Health* (NIH).^11 , 12^

Uma vez que a AT é uma condição rara e típica em adultos jovens, estudos descrevendo populações fora dessa faixa etária começaram a ser desenvolvidos só nas últimas décadas, com ênfase na população jovem.^13^ Estudos descrevendo características clínicas de crianças e adolescentes com AT relataram uma alta prevalência de cefaleia, febre, perda de peso, e insuficiência cardíaca, bem como elevada proporção de hipertensão renovascular.^14 - 18^ Em descrições angiográficas, o achado mais prevalente é o envolvimento de toda a aorta, além de progressões clínica e de imagem mesmo em pacientes com doença bem controlada.^17 - 20^

Embora escassos, estudos têm sugerido que o curso da AT varia entre pacientes jovens e adultos.^19 , 21^ Em um estudo comparando essas duas populações, encontrou-se que o comprometimento renal, com evolução para hipertensão renovascular, é mais frequente em jovens, com estenose da artéria renal esquerda e, na AT adulta, há uma tendência de envolvimento de lesões nas artérias subclávia.^21^ Observou-se que a idade média dos pacientes no grupo com AT adulta foi de 29 anos, o que é compatível com a apresentação epidemiológica clássica da doença, sem descrição de parâmetros clínicos ou atividade da doença em outros grupos etários, principalmente em pacientes com idade avançada.

Neste contexto, até o momento, nenhum estudo avaliou comparativamente indivíduos adultos com AT em uma idade mais avançada, levantando a questão sobre as implicações e possíveis diferenças na abordagem dessa população, especialmente se considerados possíveis fatores de confusão no diagnóstico e atividade da AT. Além disso, deve-se considerar que doenças como aterosclerose, osteoartrite, dor difusa, e fibromialgia são altamente prevalentes em indivíduos mais velhos.^22 , 23^ Assim, o objetivo do presente estudo foi avaliar, por grupo etário, pacientes adultos com AT quanto à atividade da doença, tratamento farmacológico (por exemplo, uso de glicocorticoides e agentes imunossupressores/ biológicos) e possíveis complicações crônicas da doença.

## Métodos

Este estudo transversal, retrospectivo, do tipo coorte foi conduzido em dois centros entre 2019 e 2021. A amostra foi selecionada de maneira não probabilística (amostragem por conveniência) por recrutamento ativo. Os pacientes tinham idade superior a 18 anos e eram acompanhados em dois ambulatórios terciários de reumatologia. Todos os pacientes preencheram os critérios de classificação para AT do *American College of Rheumatology* de 1990:^24^ (i) idade no início da doença ≤ 40 anos, (ii) claudicação de extremidades, (iii) pulso braquial reduzido, (iv) diferença na pressão arterial > 10 mmHg, (v) ruído na artéria subclávia ou aorta, e (vi) achados de imagem anormais (angiografia ou tomografia computadorizada convencional ou ressonância magnética). A AT foi confirmada se três ou mais desses seis critérios estivessem presentes.

Este estudo foi aprovado pelo comitê de ética local (CAAE 89386618.0.1001.0068), e todos os pacientes assinaram o termo de consentimento.

Para a análise transversal, os dados foram obtidos por meio de um questionário clínico e epidemiológico, e entrevistas para determinar o estado do paciente. Ainda, como análise retrospectiva, dados de todos os pacientes dos 12 últimos meses anteriores às dadas das entrevistas foram coletados dos prontuários eletrônicos dos pacientes.

Os seguintes dados foram avaliados – características gerais dos pacientes (sexo, idade no diagnóstico, duração da doença, etnia, e período de acompanhamento ambulatorial), comorbidades (hipertensão arterial, diabetes mellitus, dislipidemia, fibromialgia e tabagismo), dados laboratoriais (velocidade de hemossedimentação e proteína C reativa), características de imagens de angiotomografia (classificação angiográfica de Hata de AT^25^) ( Tabela Suplementar 4 ), tratamento (uso de glicocorticoides, agentes imunossupressores ou biológicos), status da doença (escore de atividade da arterite de Takayasu indiano – ITAS2010 *Indian Takayasu Clinical Activity Score* ), e atividade da doença (ITAS2010 ≥2)^11^ ( Tabela Suplementar 5 ), dano da doença (índice de extensão da doença para a AT – DEI.Tak, *disease extent index for Takayasu’s arteritis* )^26^ ( Tabela Suplementar 6 ), e status funcional (questionário de avaliação da saúde - HAQ, *Health Assessment Questionnaire* ).^27^

Os pacientes foram divididos em quatro quartis de acordo com idade atual, e comparados quanto as variáveis descritas.

### Análise estatística

A distribuição das variáveis foi avaliada pelo teste de Shapiro-Wilk. As variáveis contínuas sem distribuição normal foram expressas em mediana (intervalo interquartil) e variáveis categóricas em números e frequências (%). As variáveis categóricas foram comparadas pelo teste do qui-quadrado ou o teste exato de Fisher, de acordo com a distribuição dos dados e presunções estatísticas. Análise inferencial das variáveis (analisadas por idade) sem distribuição normal foi realizada pelo teste de Kruskal-Wallis e, quando uma diferença significativa era observada, o teste de Dunn para comparações múltiplas pareadas foi realizado. Um valor de p<0,05 foi considerado significativo. Todas as análises foram realizadas utilizando-se o programa SPSS 22.0 (IL, Armonk, NY, EUA).

## Resultados

No presente estudo, 66 pacientes com AT foram avaliados, 94,9% eram mulheres, e 73,7% e 26,3% eram de etnia branca e negra, respectivamente. Desses, 16 encontravam-se no primeiro quartil (Q1: 22 - 36 anos), 18 no segundo (Q2: 37 - 42 anos), 17 no terceiro (Q3: 43 - 49 anos) e 15 no quarto (Q4: 51 - 66 anos). Características demográficas gerais (idade, etnia, sexo, e duração da doença) dos quatro grupos, bem como os parâmetros relacionados ao status atual da doença estão descritas na Tabela 1 .

**Tabela 1:** Características demográficas e status da doença dos pacientes com arterite de Takayasu de acordo com a distribuição da idade (quartis)

	Q1 (n=16)	Q2 (n=18)	Q3 (n=17)	Q4 (n=15)	Valor p	Post-hoc (valor p)					

						Q1 *vs* Q2	Q1 *vs* Q3	Q1 *vs* Q4	Q2 *vs* Q3	Q2 *vs* Q4	Q3 *vs* Q4
**Dados demográficos**											
Idade atual (anos)	32,5 (28,5-33,0)	40,5 (40,0-41,7)	47,0 (45,0-48,0)	58,0 (53,0-60,5)	<0,001	<0,001	<0,001	<0,001	<0,001	<0,001	<0,001
Idade no diagnóstico (anos)	23,0 (19,7-25,2)	31,0 (27,7-37,0)	32,0 (27,7-39,0)	44,0 (30,5-47,0)	<0,001	0,006	0,004	<0,001	>0,999	0,007	0,011
Etnia branca	11 (68,8)	10 (55,6)	9 (52,9)	10 (66,7)	0,732	-	-	-	-	-	-
Sexo feminino	15 (93,8)	16 (88,9)	16 (94,1)	14 (93,3)	>0,999	-	-	-	-	-	-
Duração da doença (anos)	8,0 (3,7-9,0)	10,0 (4,2-11,7)	17,0 (8,0-19,0)	16,0 (11,5-21,0)	<0,001	0,962	0,017	0,001	0,049	0,003	0,724
**Status atual da doença**											
Escore ITAS2010	0,00 (0,0-0,0)	0,00 (0,0-0,0)	0,00 (0,0-0,0)	0,00 (0,0-0,0)	0,966	-	-	-	-	-	-
Velocidade de hemossedimentação (mm/1st hora)	9,5 (4,7-22,0)	15,5 (10,7-24,7)	14,0 (9,0-18,0)	21,5 (11,2-28,0)	0,353	-	-	-	-	-	-
PCR (mg/L)	1,7 (0,8-6,8)	3,0 (1,6-7,7)	2,6 (1,3-4,0)	3,8 (1,0-5,1)	0,641	-	-	-	-	-	-
**Status da doença (últimos 12 meses)**											
Escore ITAS2010	0,00 (0,0-0,0)	0,00 (0,0-0,0)	0,00 (0,0-0,0)	0,00 (0,0-0,0)	0,771	-	-	-	-	-	-
Atividade ITAS2010 (≥ 2 pontos)	1 (6,2)	3 (16,7)	3 (17,6)	2 (13,3)	0,836	-	-	-	-	-	-
DEI-Takayasu	3,0 (2,0-4,0)	3,0 (2,0-3,0)	2,0 (1,0-4,0)	2,0 (1,0-2,5)	0,477	-	-	-	-	-	-
Novos sintomas relacionados à AT	5 (31,2)	7 (41,2)	2 (11,8)	4 (26,7)	0,280	-	-	-	-	-	-

*Dados expressos em mediana (intervalor interquartil) ou frequência (%). Q1= 22-36 anos; Q2= 37-42 anos; Q3= 43-49 anos; Q4 = 51-66 anos. PCR: Proteína C-reativa; DEI: índice de extensão da doença; ITAS: Indian Takayasu Activity Score; AT: arterite de Takayasu.*

A atividade da doença nos últimos 12 meses (ITAS2010, DEI-TAK, novos sintomas e complicações relacionados à AT) está apresentada na Tabela 1 .

Não foram observados casos de infarto agudo do miocárdio ou revascularização coronária durante o período de análise. Entretanto, dois pacientes apresentaram ataque isquêmico transitório (49 e 59 anos de idade) e seis pacientes apresentaram desenvolvimento ou piora da claudicação de membros (idades 29, 34, 36, 40, 40 e 59 anos). Não houve ocorrência de óbito.

Além da idade e da duração da doença, que foi mais alta no Q4 que nos demais grupos (p<0,001 e p=0,001, respectivamente), todos os outros parâmetros apresentados na Tabela 1 eram comparáveis entre os quatro quartis.

**Figura 1:**
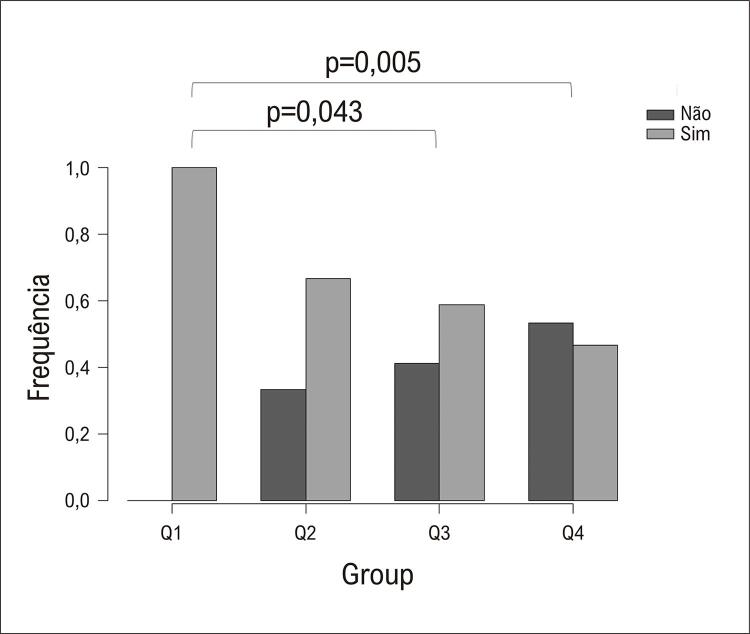
Frequência de pacientes com arterite de Takayasu utilizando drogas imunossupressoras ou biológicas, de acordo com os quartis de idade: Q1 (22-36 anos), Q2 (37-42 anos), Q3 (43-49 anos), e Q4 (51-66 anos).

Em relação aos padrões angiográficos segundo a classificação de Hata, não foram observadas diferenças entre os grupos ( Tabela 2 ), nem diferenças em comorbidades, hábitos ou fadiga. Contudo, pacientes no quarto quartil ( *i.e* ., mais velhos), em comparação aos pacientes no segundo quartil, mostraram capacidade reduzida para realizar atividades diárias, ou seja, relacionadas ao status funcional (p=0,033). Em relação ao tratamento e idade atual, um menor número de pacientes mais velhos usava prednisona (Q1: 43,8%, Q2: 33,3%, Q3: 11,8%, e Q4: 6,7%; p=0,049), embora nenhuma diferença tenha sido detectada na análise post-hoc. Diferenças significativas foram observadas no uso de agentes imunossupressores e biológicos (Q1:100,0%, Q2:66,7%, Q3:58,8%, e Q4:46,7%) entre os grupos Q1 *versus* Q3, e Q1 *versus* Q4, nas análises post-hoc ( Tabela 3 e Figura 1 ).

**Tabela 2:** Classificação angiográfica, comorbidades, fadiga, e capacidade funcional dos pacientes com arterite de Takayasu de acordo com a distribuição da idade (quartis)

	Q1 (n=16)	Q2 (n=18)	Q3 (n=17)	Q4 (n=15)	Valor p	Post-hoc (Valor p)					

						Q1 *vs* Q2	Q1 *vs* Q3	Q1 *vs* Q4	Q2 *vs* Q3	Q2 *vs* Q4	Q3 *vs* Q4
**Classificação angiográfica**											
Hata I	2 (12,5)	2 (11,1)	1 (5,9)	1 (6,7)	0,945	-	-	-	-	-	-
Hata IIa	1 (6,2)	2 (11,1)	1 (5,9)	3 (20,0)	0,588	-	-	-	-	-	-
Hata IIb	2 (12,5)	3 (16,7)	3 (17,6)	1 (6,7)	0,863	-	-	-	-	-	-
Hata III	0	2 (11,1)	1 (5,9)	1 (6,7)	0,786	-	-	-	-	-	-
Hata IV	2 (12,5)	1 (5,6)	1 (5,9)	1 (6,7)	0,864	-	-	-	-	-	-
Hata V	9 (56,2)	8 (44,4)	10 (58,8)	8 (53,3)	0,842	-	-	-	-	-	-
**Comorbidades e hábitos**						-	-	-	-	-	-
Hipertensão	12 (75,0)	15 (83,3)	14 (82,4)	13 (86,7)	0,900	-	-	-	-	-	-
Dislipidemia	6 (37,5)	10 (55,6)	13 (76,5)	12 (80,0)	0,052	-	-	-	-	-	-
Diabetes mellitus	0	1 (5,6)	2 (11,8)	3 (20,0)	0,213	-	-	-	-	-	-
Fibromialgia	1 (6,2)	3 (16,7)	2 (11,8)	2 (13,3)	0,887	-	-	-	-	-	-
Fumante atual	0	1 (5,6)	1 (5,9)	1 (6,7)	0,893	-	-	-	-	-	-
Ex-fumante	0	3 (16,7)	3 (17,6)	2 (13,3)	0,356	-	-	-	-	-	-
EAV fadiga (0-10 cm)	5,0 (2,0-6,6)	4,5 (2,0-6,0)	5,0 (2,0-6,0)	7,0 (4,5-8,0)	0,231	-	-	-	-	-	-
HAQ (0,00-3,00)	0,6 (0,2-0,9)	0,4 (0,0-1,0)	0,6 (0,2-1,0)	1,0 (0,6-1,7)	0,038	0,953	>0,999	0,129	0,944	0,033	0,126

*Dados expressos em mediana (intervalor interquartil) ou frequência (%). Q1= 22-36 anos; Q2= 37-42 anos; Q3= 43-49 anos; Q4 = 51-66 anos. EAV: Escala Analógica Visual; HAQ: Health Assessment Questionnaire.*

**Tabela 3:** Tratamento atual dos pacientes com arterite de Takayasu de acordo com a distribuição da idade (quartis)

	Q1 (n=16)	Q2 (n=18)	Q3 (n=17)	Q4 (n=15)	Valor p	Post-hoc (Valor p)					

						Q1 *vs* Q2	Q1 *vs* .Q3	Q1 *vs* .Q4	Q2 *vs.* Q3	Q2 *vs.* Q4	Q3 *vs.* Q4
**Prednisona**											
Em uso atual (%)	7 (43,8)	6 (33,3)	2 (11,8)	1 (6,7)	0,049	>0,999	0,290	0,220	0,690	0,380	>0,999
Dose (mg/dia)	0,0 (0,0-5,6)	0,0 (0,0-6,8)	0,0 (0,0-0,0)	0,0 (0,0-0,0)	0,026	>0,999	0,110	0,140	0,200	0,220	>0,999
< 5 mg/dia	12 (75,0)	13 (72,2)	16 (94,1)	14 (93,3)	0,197	-	-	-	-	-	-
5 - 10 mg/dia	2 (12,5)	3 (16,7)	1 (5,9)	1 (6,7)	0,791	-	-	-	-	-	-
> 10 mg/dia	2 (12,5)	2 (11,1)	0	0	0,291	-	-	-	-	-	-
**Drogas imunossupressoras ou biológicas**											
Em uso atual (qualquer medicamento)	16 (100,0)	12 (66,7)	10 (58,8)	7 (46,7)	0,004	0,119	0,043	0,005	>0,999	>0,999	>0,999
Azatioprina	6 (37,5)	4 (22,2)	1 (5,9)	1 (6,7)	0,083	-	-	-	-	-	-
Metotrexato	5 (31,2)	6 (33,3)	5 (29,4)	1 (6,7)	0,258	-	-	-	-	-	-
Micofenolato de mofetila	0	0	1 (5,9)	1 (6,7)	0,470	-	-	-	-	-	-
Leflunomida	5 (31,2)	1 (5,6)	3 (17,6)	3 (20,0)	0,280	-	-	-	-	-	-
Tocilizumabe	2 (12,5)	1 (5,6)	0	0	0,348	-	-	-	-	-	-
Rituximabe	0	1 (5,6)	0	0	>0,999	-	-	-	-	-	-
Infliximabe	3 (18,8)	1 (5,6)	2 (11,8)	1 (6,7)	0,641	-	-	-	-	-	-
Outros	0	3 (16,7)	1 (5,9)	1 (6,7)	0,359	-	-	-	-	-	-

*Dados expressos em mediana (intervalor interquartil) ou frequência (%). Q1= 22-36 anos; Q2= 37-42 anos; Q3= 43-49 anos; Q4 = 51-66 anos.*

A figura central mostra um resumo gráfico ilustrando a história natural dos pacientes com AT de acordo com a idade e o tipo de doença vascular/sintomas clínicos.

**Figura Central:**
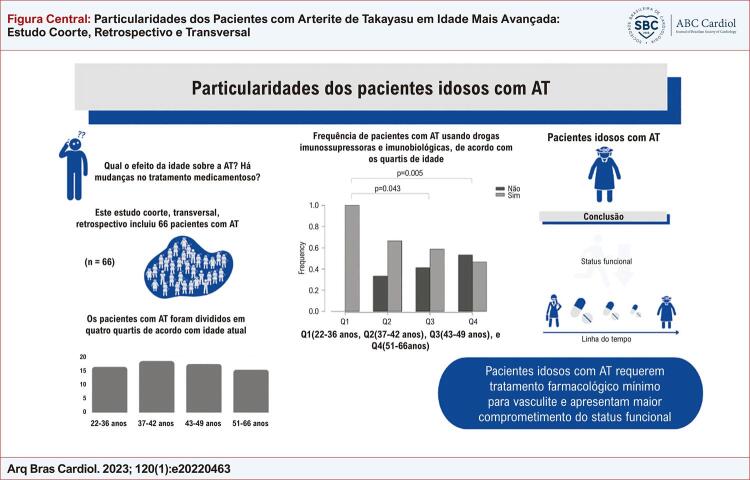
Particularidades dos Pacientes com Arterite de Takayasu em Idade Mais Avançada: Estudo Coorte, Retrospectivo e Transversal

AT: Arterite de Takayasu.

## Discussão

Este é o primeiro estudo comparando pacientes com AT, considerando o grupo etário e possíveis progressos e sequelas do tratamento durante o processo de envelhecimento nessa população. Nossos resultados indicam que pacientes idosos têm menos necessidade de tratamento farmacológico (glicocorticoides e agentes imunossupressores). Além disso, pacientes idosos com AT têm maior comprometimento em seu status funcional.

Uma das principais vantagens de nosso estudo foi a possibilidade de avaliar a população em dois momentos distintos, facilitando a avaliação contínua e progressiva dos parâmetros estudados. Ainda, uma vez que esse foi um estudo conduzido em dois centros, conseguimos recrutar um número maior de pacientes, resultando em uma amostra mais representativa da realidade, e com menos vieses.

Em concordância com as tendências epidemiológicas da doença, as mulheres jovens eram predominantes em nossa população.^2^Ainda, a etnia branca era a mais prevalente, o que está de acordo com as tendências globais da população brasileira.^28^ A duração da doença mostrou uma correlação positiva na comparação entre os quartis, mas esse foi um achado esperado para os indivíduos com idade mais avançada.

Um estudo mostrou que a população jovem com AT apresentou menores taxas de remissão que pacientes adultos, mas não que pacientes idosos, mesmo com frequências similares de terapia imunossupressora. Isso sugere um pior prognóstico nos jovens,^21^ ou mesmo uma tendência para completa remissão nos pacientes com idade mais avançada. Contudo, em relação ao status da doença, havia poucos pacientes com atividade segundo ITAS2010 em nosso estudo tanto no basal, como na avaliação retrospectiva de 12 meses.

Biomarcadores de atividade séricos, como a taxa de hemossedimentação e proteína C-reativa, são comumente usados como indicadores de atividade da doença na prática clínica,^29^ e a ausência de um critério para defini-la é uma das principais dificuldades no manejo desses pacientes. Em nosso estudo, os índices inflamatórios não foram significativamente diferentes entre os quartis. Outros estudos mostraram que nenhum teste sanguíneo específico, incluindo testes inflamatórios, podem avaliar, com segurança, a atividade da doença em comparação aos achados histopatológicos.^13^

Quanto às características angiográficas, o tipo V foi o tipo de envolvimento mais frequente em todos os quartis, sem diferença significativa para o grupo de idade mais avançada. Esses achados estão de acordo com um estudo brasileiro que relatou uma prevalência mais alta do tipo V (66,7%).^28^

Em relação aos níveis de fadiga crônica, apesar das diferenças consideráveis entre os grupos, não houve diferenças significativas entre os grupos de pacientes mais velhos e os de pacientes mais jovens. Contudo, o HAQ revelou que o comprometimento do status funcional foi mais comum nos indivíduos com idade mais avançada que nos mais jovens.

Esse resultado pode ser correlacionado com idade avançada e uma maior prevalência de comorbidades cardiovasculares, tais como hipertensão sistêmica e dislipidemia, além de um estilo de vida sedentário. Um estudo brasileiro mostrou que pacientes com AT apresentaram menor força muscular, capacidade aeróbica reduzida, aumento de tecido adiposo visceral, relação cintura-quadril aumentada, além de uma capacidade reduzida para caminhada e estilo de vida sedentário, corroborando nossos achados de capacidade reduzida para realizar atividades diárias (HAQ). Em conjunto, esses fatores resultam em um risco cardiovascular aumentado e pior status funcional.^30^ Não podemos afirmar que esse impacto funcional tenha sido causado por um aumento na cronicidade da doença com a idade, uma vez que, como comentado acima, não houve diferença no padrão de atividade da doença atual ou prévia entre os grupos, sobretudo quando avaliamos os efeitos negativos da doença utilizando o DEI-Takayasu.

Segundo o HAQ, os pacientes com AT mais velhos apresentaram maior tendência a serem mais sintomáticos. Ainda, é possível que esses sintomas causados por comprometimento funcional tenham sido erroneamente classificados como critérios de atividade da doença, o que pode ter contaminado os resultados da avaliação clínica, e indicado uma maior necessidade para intervenções terapêuticas desnecessárias. Ainda, em um estudo, a escala HAQ serviu como um domínio para avaliação da doença em uma população com AT, uma vez que essa encontrava-se alterada independentemente da atividade da doença ou idade.^31^

Uma possível limitação deste estudo foi a seleção dos pacientes por conveniência. Contudo, enfatizamos a grande dificuldade de se realizar uma avaliação tão complexa em uma população com AT, a qual é considerada uma doença rara. Ainda, incluímos somente pacientes atendidos em centros terciários de reumatologia; apesar da possibilidade de maior gravidade, esses pacientes apresentaram níveis mínimos de atividade da doença de acordo com os padrões ITAS2020. Tal fato provavelmente deve-se mais à maior experiência da parte das equipes médicas envolvidas em alcançar a remissão da doença, que à ideia de que o curso natural da doença com a idade resulta em menor necessidade de medicação.

## Conclusões

Pacientes idosos com AT requerem mínima intervenção medicamentosa e sofrem maior impacto sobre o status funcional, requerendo uma classificação cuidadosa da atividade da doença, e possíveis mudanças no tratamento medicamentoso devido a potenciais erros diagnósticos. Com base em nossos resultados e na escassez de estudos sobre o assunto, mais estudos são necessários envolvendo pacientes idosos com AT.
