# Supplementary material for: Particularidades dos Pacientes com Arterite de Takayasu em Idade Mais Avançada: Estudo Coorte, Retrospectivo e Transversal
Source: Arq Bras Cardiol. 2022 Dec 20;120(1):e20220463. [Article in Portuguese] doi: 10.36660/abc.20220463 (PMC9833251; doi:10.36660/abc.20220463)
Supplement: Translation

# Translation: Characteristics of Older Patients with Takayasu’s Arteritis: A Two-Center, Cross-Sectional, Retrospective Cohort Study

**Authors:** João Calvino Soares de Oliveira, Alexandre Moura dos Santos, Mariana Freitas de Aguiar, Jucier Gonçalves, Alexandre Wagner Silva de Souza, Rosa Maria R. Pereira, Samuel Katsuyuki Shinjo

**Affiliations:** 1 Brazil Divisão de Reumatologia – Hospital das Clínicas HCFMUSP – Faculdade de Medicina – Universidade de São Paulo, São Paulo, SP – Brazil; 2 Brazil Divisão de Reumatologia – Universidade Federal de São Paulo – UNIFESP,São Paulo, SP – Brazil; † In memorium

**Keywords:** Aging, Systemic Vasculitis, Aortic Diseases

## Abstract

**Background:**

Few studies have assessed elderly patients with Takayasu’s arteritis (TAK).

**Objectives:**

To evaluate the progression of TAK in different age groups and its possible effects on drug treatment and disease activity.

**Methods:**

This cross-sectional and retrospective cohort study included 66 TAK patients. Patients were interviewed and data of the 12 preceding months were collected from electronic medical records. The patients were divided into four quartiles according to current age and compared for clinical and laboratory data, treatment, comorbidities, disease status, and functional status. Statistical significance was set at p<0.05.

**Results:**

The groups were Q1(22-36 years, n=16), Q2(37-42 years, n=18), Q3(43-49 years, n=17), and Q4(51-66 years, n=15). The frequency of patients with disease activity, fatigue, comorbidities and vascular impairments, and the TAK disease extent index were also comparable between the groups. With age, disease duration was longer (p=0.001), fewer patients used prednisone (Q1:43.8%, Q2:33.3%, Q3:11.8%, and Q4:6.7%; p=0.049) and immunosuppressive drugs [Q1:100.0%, Q2:66.7%, Q3:58.8%, and Q4:46.7%; Q1 versus Q3 (p=0.043), and Q1 versus Q4 (p=0.005) in post-hoc analyses], and patients had greater functional status impairment (Q2 versus Q3, p=0.003). In addition, the levels of disease damage, new TAK symptoms, and complications in the preceding 12 months were not different between the groups.

**Conclusions:**

Older patients with TAK require minimal drug treatment, and have greater impairment of functional status, which may be attributed to aging-related factors.

## Introduction

Takayasu’s arteritis (TAK) is a primary systemic vasculitis that preferentially affects large-caliber vessels, such as the aorta and its proximal branches. Disease onset occurs most commonly in women younger than 40.^1 , 2^

TAK is characterized clinically by alternating periods of activity and remission, directly reflecting the various inflammatory states of the affected vessels.^3 - 5^About 20% of patients have a monophasic and self-limited course, with constitutional symptoms that may not have clinical repercussions. However, the remainder may have progressive vascular inflammation or a severe remitting/recurrent course, with clinical manifestations varying according to the affected vascular territory, limb claudication, syncope, thoracic pain, renovascular hypertension, and reduced/absence of pulses.^6 - 8^

However, in clinical practice, characterizing disease activity is often difficult, because inflammation of the vessel walls does always reflect or follow other systemic inflammation manifestations or increased acute phase reactants.^9^On the other hand, a detailed history, physical evaluation, and new imaging findings can help define the disease state.^10^Also, despite their limitations, there are criteria to assess disease activity in TAK, such as the Indian Takayasu Clinical Activity Score (ITAS2010) and the National Institute of Health (NIH) score.^11 , 12^

Since TAK is a rare and typical condition in young adults, studies describing populations outside this age range have only started to be developed in recent decades, with emphasis on the juvenile population.^13^In studies describing clinical characteristics of children and adolescents with TAK reported a high prevalence of headache, fever, weight loss, arthritis, and heart failure, as well as a high proportion of renovascular hypertension.^14 - 18^In angiographic descriptions, the most prevalent finding is pan-aortic involvement, in addition to clinical and imaging progression even in well-controlled disease.^17 - 20^

Although studies are scarce, it is suggested that the TAK course vary between juvenile and adult TAK.^19 , 21^In a study comparing these two populations, it was found that renal involvement, with progression to renovascular hypertension, is more common in young patients, in which stenosis of the left renal artery is more frequent, whereas in adult TAK, there is a tendency of involvement of lesions in subclavian arteries.^21^It was found that the mean age of patients in the adult TAK group was 29 years, which is compatible with the classic epidemiological presentation of the disease, with no description of clinical parameters or disease activity in other age groups, especially in patients with TAK with advanced age.

In this context, no studies to date have comparatively evaluated adult individuals with TAK at an older age, raising the question of the implications and possible differences in the approach to this population, especially when considering possible confounders for the diagnosis and activity of TAK. In addition, it should be considered that diseases such as atherosclerosis, osteoarthritis, diffuse pain, and fibromyalgia are highly prevalent in older individuals.^22 , 23^Therefore, the aim of the present study was to assess adult TAK patients for disease activity, drug treatment ( *e.g.* , use of glucocorticoids and immunosuppressants/ biologics) and possible chronic complications of the disease by age group.

## Methods

This two-center, cross-sectional, retrospective cohort study was conducted between 2019 and 2021. The sample was selected in a non-probabilistic manner (convenience sampling) through active recruitment. Patients were aged ≥18 years and were seen at two tertiary rheumatology outpatient clinics. All patients fulfilled the 1990 American College of Rheumatology classification criteria for TAK:^24^(i) age of disease onset ≤ 40 years, (ii) claudication of extremities, (iii) decreased brachial artery pulse, (iv) blood pressure difference > 10 mmHg, (v) bruit over the subclavian arteries or aorta, and (vi) imaging (angiographic or conventional computed tomography or magnetic resonance imaging) abnormalities. TAK was confirmed if at least three of these six criteria were met.

This study was approved by the local ethics committee (CAAE 89386618.0.1001.0068), and written informed consent was obtained from all patients.

For the cross-sectional analysis, data were obtained using a clinical-epidemiological questionnaire and interviews to determine patient status. In addition, as a retrospective analysis, all patients’ data from the 12 months preceding the interview dates were obtained from the electronic medical records.

The following variables were assessed – patient general characteristics (sex, age at diagnosis, disease duration, ethnicity, and outpatient follow-up period), comorbidities (systemic arterial hypertension, diabetes mellitus, dyslipidemia, fibromyalgia, and smoking status), laboratory data (erythrocyte sedimentation rate and C-reactive protein), angiography image features (Hata’s angiographic classification of TAK^25^) ( Supplementary Table 4 ), treatment (use of glucocorticoids, immunosuppressive or immunobiological drugs), disease status (Indian Takayasu Clinical Activity Score - ITAS2010), and disease activity (ITAS2010 ≥ 2^11^) ( Supplementary Table 5 ), disease damage (disease extent index for Takayasu’s arteritis – DEI.Tak^26^) ( Supplementary - Table 6 ), and functional status (Health Assessment Questionnaire - HAQ).^27^

Patients were divided into four quartiles according to their current age and compared according to the variables described.

### Statistical analysis

The distribution of variables was assessed by the Shapiro-Wilk test. Continuous variables with non-normal distribution were expressed as median (interquartile range), and categorical variables were represented by number and frequency (%). Categorical variables were compared using the chi-square test or Fisher’s exact test, according to data distribution and statistical assumptions. Inferential analysis of variables (analyzed by age) with non-normal distribution was performed using the Kruskal-Wallis test, and when a significant difference was found, Dunn’s test for multiple pairwise comparisons was performed. A p-value <0.05 was considered significant. All analyses were performed using SPSS 22.0 statistical software (IL, Armonk, NY, USA).

## Results

In the present study, 66 TAK patients were evaluated; 94.9% of whom were women, and 73.7% and 26.3% were of white and black ethnicities, respectively. Of these, 16 were in the first quartile (Q1: 22 - 36 years), 18 in the second (Q2: 37 - 42 years), 17 in the third (Q3: 43 - 49 years) and 15 in the fourth (Q4: 51 - 66 years). General demographic characteristics (age, ethnicity, sex, and disease duration) of the four groups, as well as the parameters regarding the current disease status are shown in Table 1 .

**Table 1:** Demographic features and disease status of patients with Takayasu’s arteritis according to current age distribution (quartiles)

	Q1 (n=16)	Q2 (n=18)	Q3 (n=17)	Q4 (n=15)	p-value	Post-hoc (p-value)					

						Q1 *vs* Q2	Q1 *vs* Q3	Q1 *vs* Q4	Q2 *vs* Q3	Q2 *vs* Q4	Q3 *vs* Q4
**Demographic data**											
Current age (years)	32.5 (28.5-33.0)	40.5 (40.0-41.7)	47.0 (45.0-48.0)	58.0 (53.0-60.5)	<0.001	<0.001	<0.001	<0.001	<0.001	<0.001	<0.001
Age at disease diagnosis (years)	23.0 (19.7-25.2)	31.0 (27.7-37.0)	32.0 (27.7-39.0)	44.0 (30.5-47.0)	<0.001	0.006	0.004	<0.001	>0.999	0.007	0.011
White ethnicity	11 (68.8)	10 (55.6)	9 (52.9)	10 (66.7)	0.732	-	-	-	-	-	-
Female sex	15 (93.8)	16 (88.9)	16 (94.1)	14 (93.3)	>0.999	-	-	-	-	-	-
Disease duration (years)	8.0 (3.7-9.0)	10.0 (4.2-11.7)	17.0 (8.0-19.0)	16.0 (11.5-21.0)	<0.001	0.962	0.017	0.001	0.049	0.003	0.724
**Current disease status**											
ITAS2010 score	0.00 (0.0-0.0)	0.00 (0.0-0.0)	0.00 (0.0-0.0)	0.00 (0.0-0.0)	0.966	-	-	-	-	-	-
ESR (mm/1st hour)	9.5 (4.7-22.0)	15.5 (10.7-24.7)	14.0 (9.0-18.0)	21.5 (11.2-28.0)	0.353	-	-	-	-	-	-
CRP (mg/L)	1.7 (0.8-6.8)	3.0 (1.6-7.7)	2.6 (1.3-4.0)	3.8 (1.0-5.1)	0.641	-	-	-	-	-	-
**Disease status (last 12 months)**											
ITAS2010 score	0.00 (0.0-0.0)	0.00 (0.0-0.0)	0.00 (0.0-0.0)	0.00 (0.0-0.0)	0.771	-	-	-	-	-	-
ITAS2010 activity (≥ 2 points)	1 (6.2)	3 (16.7)	3 (17.6)	2 (13.3)	0.836	-	-	-	-	-	-
DEI-Takayasu	3.0 (2.0-4.0)	3.0 (2.0-3.0)	2.0 (1.0-4.0)	2.0 (1.0-2.5)	0.477	-	-	-	-	-	-
New symptoms related to TAK	5 (31.2)	7 (41.2)	2 (11.8)	4 (26.7)	0.280	-	-	-	-	-	-

*Data expressed as median (interquartile range) or frequency (%). Q1= 22-36 years; Q2= 37-42 years; Q3= 43-49 years; Q4 = 51-66 years. CRP: C-reactive protein; DEI: Disease Extent Index; ESR: erythrocyte sedimentation rate; ITAS: Indian Takayasu Activity Score; TAK: Takaysu’s arteritis.*

The disease activity in the last 12 months (ITAS2010, DEI-TAK, new symptoms and new complications related to TAK) is presented in Table 1 .

No cases of acute myocardial infarction or coronary revascularization were observed during the analysis period. However, there were two patients with transient ischemic attack (aged 49 and 59 years) and six patients had new or worsened intermittent limb claudication (29, 34, 36, 40, 40, and 59 years of age). There was no case of deaths.

In addition to age and disease duration, which were higher in Q4 than in the other groups (p<0.001 and p=0.001, respectively), all other parameters shown in Table 1 were comparable between the four quartiles.

Regarding the angiographic patterns according to Hata’s classification, there were no significant differences between the groups ( Table 2 ), or differences in comorbidities, habits, and fatigue. However, patients in the Q4 group ( *i.e* ., older) when compared to the Q2 group showed reduced ability to perform activities of daily living, that is, functional status (p=0.033). Regarding treatment and current age, fewer older patients used prednisone (Q1: 43.8%, Q2: 33.3%, Q3: 11.8%, and Q4: 6.7%; p=0.049), although no difference was detected in the post-hoc analysis. Significant differences in immunosuppressive or immunobiological drugs (Q1:100.0%, Q2:66.7%, Q3:58.8%, and Q4:46.7%) were found between groups Q1 *versus* Q3, and Q1 *versus* Q4 in the post-hoc analyses ( Table 3 and Figure 1 ).

**Table 2:** Angiographic classification, comorbidities, fatigue and functional capacity of patients with Takayasu’s arteritis according to current age distribution (quartiles)

	Q1 (n=16)	Q2 (n=18)	Q3 (n=17)	Q4 (n=15)	p-value	Post-hoc (p-value)					

						Q1 *vs* Q2	Q1 *vs* Q3	Q1 *vs* Q4	Q2 *vs* Q3	Q2 *vs* Q4	Q3 *vs* Q4
**Angiographic classification**											
Hata I	2 (12.5)	2 (11.1)	1 (5.9)	1 (6.7)	0.945	-	-	-	-	-	-
Hata IIa	1 (6.2)	2 (11.1)	1 (5.9)	3 (20.0)	0.588	-	-	-	-	-	-
Hata IIb	2 (12.5)	3 (16.7)	3 (17.6)	1 (6.7)	0.863	-	-	-	-	-	-
Hata III	0	2 (11.1)	1 (5.9)	1 (6.7)	0.786	-	-	-	-	-	-
Hata IV	2 (12.5)	1 (5.6)	1 (5.9)	1 (6.7)	0.864	-	-	-	-	-	-
Hata V	9 (56.2)	8 (44.4)	10 (58.8)	8 (53.3)	0.842	-	-	-	-	-	-
**Comorbidities and habits** -											
Hypertension	12 (75.0)	15 (83.3)	14 (82.4)	13 (86.7)	0.900	-	-	-	-	-	-
Dyslipidemia	6 (37.5)	10 (55.6)	13 (76.5)	12 (80.0)	0.052	-	-	-	-	-	-
Type 2 diabetes mellitus	0	1 (5.6)	2 (11.8)	3 (20.0)	0.213	-	-	-	-	-	-
Fibromyalgia	1 (6.2)	3 (16.7)	2 (11.8)	2 (13.3)	0.887	-	-	-	-	-	-
Current smoker	0	1 (5.6)	1 (5.9)	1 (6.7)	0.893	-	-	-	-	-	-
Ex-smoker	0	3 (16.7)	3 (17.6)	2 (13.3)	0.356	-	-	-	-	-	-
VAS’ fatigue (0-10 cm)	5.0 (2.0-6.6)	4.5 (2.0-6.0)	5.0 (2.0-6.0)	7.0 (4.5-8.0)	0.231	-	-	-	-	-	-
HAQ (0.00-3.00)	0.6 (0.2-0.9)	0.4 (0.0-1.0)	0.6 (0.2-1.0)	1.0 (0.6-1.7)	0.038	0.953	>0.999	0.129	0.944	0.033	0.126

*Data expressed as median (interquartile range) or frequency (%). Q1= 22-36 years; Q2= 37-42 years; Q3= 43-49 years; Q4 = 51-66 years. HAQ: Health Assessment Questionnaire; VAS: Visual analogic scale.*

**Table 3:** Current treatment of patients with Takayasu’s arteritis according to current age distribution (quartiles)

	Q1 (n=16)	Q2 (n=18)	Q3 (n=17)	Q4 (n=15)	p-value	Post-hoc (p-value)					

						Q1 *vs* Q2	Q1 *vs* .Q3	Q1 *vs* .Q4	Q2 *vs.* Q3	Q2 *vs.* Q4	Q3 *vs.* Q4
**Prednisone**											
Current use (%)	7 (43.8)	6 (33.3)	2 (11.8)	1 (6.7)	0.049	>0.999	0.290	0.220	0.690	0.380	>0.999
Dose (mg/day)	0.0 (0.0-5.6)	0.0 (0.0-6.8)	0.0 (0.0-0.0)	0.0 (0.0-0.0)	0.026	>0.999	0.110	0.140	0.200	0.220	>0.999
< 5 mg/day	12 (75.0)	13 (72.2)	16 (94.1)	14 (93.3)	0.197	-	-	-	-	-	-
5 - 10 mg/day	2 (12.5)	3 (16.7)	1 (5.9)	1 (6.7)	0.791	-	-	-	-	-	-
> 10 mg/day	2 (12.5)	2 (11.1)	0	0	0.291	-	-	-	-	-	-
**Immunossupressive or immunobiological drugs**											
Current use (any drug)	16 (100.0)	12 (66.7)	10 (58.8)	7 (46.7)	0.004	0.119	0.043	0.005	>0.999	>0.999	>0.999
Azathioprine	6 (37.5)	4 (22.2)	1 (5.9)	1 (6.7)	0.083	-	-	-	-	-	-
Methotrexate	5 (31.2)	6 (33.3)	5 (29.4)	1 (6.7)	0.258	-	-	-	-	-	-
Mycophenolate mofetil	0	0	1 (5.9)	1 (6.7)	0.470	-	-	-	-	-	-
Leflunomide	5 (31.2)	1 (5.6)	3 (17.6)	3 (20.0)	0.280	-	-	-	-	-	-
Tocilizumab	2 (12.5)	1 (5.6)	0	0	0.348	-	-	-	-	-	-
Rituximab	0	1 (5.6)	0	0	>0.999	-	-	-	-	-	-
Infliximab	3 (18.8)	1 (5.6)	2 (11.8)	1 (6.7)	0.641	-	-	-	-	-	-
Others	0	3 (16.7)	1 (5.9)	1 (6.7)	0.359	-	-	-	-	-	-

*Data expressed as median (25^*th*^-75^*th*^) or frequency (%). Q1= 22-36 years; Q2= 37-42 years; Q3= 43-49 years; Q4 = 51-66 years.*

**Figure 1:** Frequency of Takayasu’s arteritis patients using immunosuppressive or immunobiological drugs, according to the age quartiles: Q1 (22-36 years old), Q2 (37-42 years old), Q3 (43-49 years old), and Q4 (51-66 years old).

The central figure shows a graphical abstract illustrating the natural history of patients with TAK according to age and the type of vascular disease/clinical symptoms.

**Central Illustration:** Characteristics of Older Patients with Takayasu’s Arteritis: A TwoCenter, Cross-Sectional, Retrospective Cohort Study

*TAK: Takayasu’s arteritis.*

## Discussion

This is the first study to compare patients with TAK, considering the age group and possible treatment progressions and sequelae during the aging process in this population. Our findings indicate that elderly patients have less need for pharmacological treatment (glucocorticoids and immunosuppressive drugs). In addition, elderly patients with TAK have greater impairment in their functional status.

One of the main advantages of our study was the possibility of evaluating the population at two distinct time points, facilitating the continuous and progressive assessment of the studied parameters. Moreover, because this was a two-center study, we were able to recruit a larger number of participants, resulting in a sample that was more representative of reality and had fewer biases.

In line with the disease’s epidemiological trends, young women were predominant in our population.^2^In addition, White ethnicity was more prevalent, which matches overall trends in the Brazilian population.^28^The duration of disease revealed a positive correlation in the comparison between quartiles, but this was an expected finding for older individuals.

A study demonstrated that the young population with TAK had lower remission rates than adult, but not than older TAK patients, even with similar frequencies of immunosuppressive therapy, suggesting a worse prognosis among the young,^21^or even a trend towards complete remission in older patients. However, regarding disease status, there were very few patients with ITAS2010 activity in our study, both at baseline and at the 12-month retrospective evaluation.

Serum activity biomarkers, such as erythrocyte sedimentation rate and C-reactive protein, are commonly used as indicators of disease activity in clinical practice,^29^and the absence of a criterion for defining it is one of the main difficulties in the management of these patients. In our study, the inflammatory indices were not significantly different between the quartiles. Other studies have shown that no specific blood tests, including inflammatory tests, can reliably assess disease activity compared to histopathological findings.^13^

Regarding angiographic features, type V was the most frequent type of involvement in all quartile groups, with no significant difference for the older population. These findings are consistent with a Brazilian study that reported a higher prevalence of the type V (66.7%).^28^

With respect to the levels of chronic fatigue, despite considerable differences between the groups, there were no significant differences between older patient groups and the others. However, the HAQ revealed that functional status impairment was more common in the older groups than in younger ones.

This result can be correlated with advanced age and a higher prevalence of cardiovascular comorbidities, such as systemic arterial hypertension and dyslipidemia, in addition to a sedentary lifestyle. A Brazilian study showed that patients with TAK had lower muscle strength, reduced aerobic capacity, increased visceral adipose tissue, higher waist-to-hip ratio, in addition to lower walking capacity and sedentary lifestyle, corroborating our findings regarding reduced ability to perform activities of daily living (HAQ). Taken together, these factors result in an increased cardiovascular risk and lower functional status.^30^We cannot assert that this functional impact was caused by an increase in the chronicity of the disease with aging because, as noted above, there was no difference in the pattern of current or previous disease activity between the quartiles, especially when we evaluated the negative effects of the disease using the DEI-Takayasu.

According to the HAQ, older TAK patients showed a greater tendency to be more symptomatic. In addition, their symptoms, which were generated by functional impact, may have been misclassified as disease activity criteria, which may have contaminated the clinical evaluation results and indicated a greater need for (unnecessary) therapeutic intervention. In addition, in one study, the HAQ scale served as a domain for disease assessment in a population with TAK because it was altered in this population regardless of disease activity or age.^31^

A possible limitation of this study was the selection of patients by convenience. However, we emphasize the great difficulty of performing such complex evaluation in a population with TAK, which is considered a rare disease. Moreover, we included only participants from tertiary rheumatology centers who, despite the possibility of increased case severity, had minimum levels of disease activity according to the ITAS2010 standards. This probably implies greater experience on the part of the medical teams involved in achieving disease remission, rather than the idea that the natural course of the disease with aging results in less need for medication.

## Conclusions

Elderly patients with TAK require minimal drug intervention and experience greater impairment of functional status, requiring a careful classification of disease activity, and possible changes in drug treatment due to potential misdiagnosis. Based on our results and on the scarcity of studies on the subject, further research focusing on older patients with TAK is needed.
